# The impact of early bilingualism on controlling a language learned late: an ERP study

**DOI:** 10.3389/fpsyg.2013.00815

**Published:** 2013-11-05

**Authors:** Clara D. Martin, Kristof Strijkers, Mikel Santesteban, Carles Escera, Robert J. Hartsuiker, Albert Costa

**Affiliations:** ^1^Basque Center on Cognition, Brain and LanguageDonostia-San Sebastian, Spain; ^2^IKERBASQUE, Basque Foundation for ScienceBilbao, Spain; ^3^Centre for Brain and Cognition, University Pompeu FabraBarcelona, Spain; ^4^Laboratoire de Psychologie Cognitive, CNRS & Université Aix-MarseilleMarseille, France; ^5^Department of Linguistics and Basque Studies, University of the Basque CountryVitoria-Gasteiz, Spain; ^6^Institute for Brain, Cognition and Behavior, University of BarcelonaBarcelona, Spain; ^7^Cognitive Neuroscience Research Group, Department of Psychiatry and Clinical Psychobiology, Faculty of Psychology, University of BarcelonaBarcelona, Spain; ^8^Department of Experimental Psychology, Ghent UniversityGhent, Belgium; ^9^Institució Catalana de Recerca i Estudis AvançatsBarcelona, Spain

**Keywords:** bilingual proficiency, language control, switch cost, N2 ERP component, LPC

## Abstract

This study asks whether early bilingual speakers who have already developed a language control mechanism to handle two languages control a dominant and a late acquired language in the same way as late bilingual speakers. We therefore, compared event-related potentials in a language switching task in two groups of participants switching between a dominant (L1) and a weak late acquired language (L3). Early bilingual late learners of an L3 showed a different ERP pattern (larger N2 mean amplitude) as late bilingual late learners of an L3. Even though the relative strength of languages was similar in both groups (a dominant and a weak late acquired language), they controlled their language output in a different manner. Moreover, the N2 was similar in two groups of early bilinguals tested in languages of different strength. We conclude that early bilingual learners of an L3 do not control languages in the same way as late bilingual L3 learners –who have not achieved native-like proficiency in their L2– do. This difference might explain some of the advantages early bilinguals have when learning new languages.

## Introduction

When learning how to speak a foreign language people need to prevent massive interference from their first language. Words and grammatical structures of the dominant language come to mind readily, making the act of producing speech in the non-dominant language a very effortful and cognitively demanding activity. Hence, a crucial ability the learner has to acquire is that of controlling verbalization in the desired language while preventing massive interference from the non-intended language. Arguably, the more effective such a language control mechanism is, the better the ability to communicate in the desired language. Here we ask whether acquiring this ability from birth affects the way a speaker handles language control in general, including control involving a weaker, late acquired language. To answer this question, we compare the language control system of a dominant and a weak late acquired language in two groups of participants: Late bilinguals and early bilinguals, all late learners of a third language. Early bilinguals are Spanish-Catalan bilingual late learners of English as an L3. Late bilinguals are Spanish native late learners of Catalan as an L3 (English being their second language)^1^. Early bilinguals are used to control L1 and L2 from birth and they use both L1 and L2 on an everyday basis. Late bilinguals started to control L1 and L2 late in life and never did it on an everyday basis. We investigate the consequence of this on the way both groups control L1 and L3.

Finding an influence of an already established bilingual language control mechanism in early bilinguals on any new language would add to the notion that knowing two languages affects learning a new one. For example, it has been shown that early bilinguals outperform monolinguals when learning a new language (Cenoz and Valencia, 1994; Lasagabaster, 2000; Sanz, 2000; Sagasta Errasti, 2003; Kaushanskaya and Marian, 2009a,b, see Cenoz, 2003 for a review). Based on these observations, it has been argued that early bilinguals learning a third language use more efficient strategies than monolinguals or late bilinguals (Cenoz, 2003).

There are also several reasons to believe that high and long-lasting command in two languages may impact the way a new language is handled by the cognitive system. It has been repeatedly shown that the need for exercising control over two languages has consequences for the development of domain-general executive control abilities. That is, early and high proficient bilingualism seems to impact both the functioning and the neural basis of general executive control mechanisms (see for instance Bialystok and Martin, 2004; Costa et al., 2009a; Abutalebi et al., 2012). Importantly, more experience in bilingualism is associated with greater advantages in general cognitive control (Luk et al., 2011). To the extent that domain-general executive control mechanisms are involved in language control (Abutalebi, 2008; Abutalebi et al., 2012), it is reasonable to assume that early bilinguals will utilize them when learning how to control a dominant and a new language.

The second reason to hypothesize differences between early and late bilinguals when learning to control a new language comes from recent imaging studies (see Abutalebi, 2008 and Indefrey, 2006 for reviews) revealing that the neural substrates of bilingual language control differ between early and late bilinguals (Wang et al., 2007; Garbin et al., 2011). The main explanation for these differences between the two types of bilingualism is that an extensive and long-lasting experience of early bilinguals with language control could presumably cause a specific development of brain structures involved in this process^2^.

In the present study, we explore whether the specific development of language control in early bilinguals (control of two dominant languages) impacts the way they control a dominant and a weak, late acquired third language. In other words, do early bilinguals benefit from the language control system they developed from birth to handle not only their two dominant languages but also a dominant and any other language? We test the hypothesis that early high proficient bilinguals benefit from the language control mechanism they have developed from birth, even when required to apply control to a third language for which they have little proficiency. Such functional differences in language control could, at least in part, be responsible for the advantage in learning a new language for early bilinguals compared to late bilinguals (learning an L3).

Until now, the comparison of early and late bilinguals controlling a dominant and a weak language was based on language switch cost patterns, but the results were not conclusive: Some studies revealed that late bilinguals show an asymmetrical switch cost: switching to the dominant language is harder than switching to the weaker language (Meuter and Allport, 1999; Costa and Santesteban, 2004). In contrast, early bilinguals also switching between a dominant and a weak language showed a symmetrical switch cost (Costa and Santesteban, 2004; Costa et al., 2006). These results have been used to conclude that language control mechanisms in early and late bilinguals may be qualitatively different, and early bilinguals can generalize such a control mechanism to any new language they learn (Costa and Santesteban, 2004). However, as recently noted by Bobb and Wodniecka (2013), one should exert caution when drawing strong conclusions from the patterns of language switch costs, because they have been shown to be less systematic than expected. For example, symmetrical switch costs are sometimes observed also in late bilinguals (e.g., Christoffels et al., 2007; Gollan and Ferreira, 2009; Verhoef et al., 2009). This finding seems problematic for the proposal that the switch cost pattern is a direct expression of the type of language control associated with a bilingual's age of acquisition. Additionally, studies that have explored the electrophysiological correlates of the language switch cost in late bilinguals have also led to somewhat inconclusive results (see Jackson et al., 2001; Christoffels et al., 2007; Verhoef et al., 2009).

Jackson and colleagues (2001) observed that the anterior N2, a component which has been associated with response selection processes and cognitive control (Folstein and Van Petten, 2008), was selectively modulated when switching into L2, but not when switching into L1. The asymmetrical modulation of the N2 parallels the asymmetrical pattern in RTs. However, subsequent studies did not replicate these results. Specifically, Christoffels et al. (2007) found a main effect of response language on the N2 (a larger negativity for L1 than L2) but this was independent of the condition (switch vs. no switch trial). In contrast, Verhoef and colleagues (2009) did find an asymmetrical modulation of the N2 component, but only when there was a relatively short interval between language cue and stimulus. When the interval was longer, so that participants had more time to prepare the response in the correct language, the modulation by switching condition disappeared.

Previous behavioral and ERP studies investigating the patterns of language switch costs did not lead to reliable empirical conclusions on how early and late bilinguals differ in the way they control a weak, late acquired language. Thus, while the present experiment also uses a language switching task (i.e., to create a need to control the output language) our main interest is not in the patterns of language switch costs, but rather in the general pattern of language control during the whole task in different types of bilinguals. Specifically, we look at how RTs and ERPs vary across different types of bilinguals (early and late bilinguals) under similar experimental circumstances.

Our study measures the electrophysiological activity in two groups of bilingual participants who performed a task that places a high demand on language control mechanisms, namely a picture naming task while switching between two languages. We compare early bilinguals performing the task in a dominant language and a much less known L3 (hereafter L1L3 EB group), and late bilinguals performing the task in their dominant language and a much less known L3 (hereafter L1L3 LB group). Note, importantly, that both groups are placed in the same experimental context, namely they have to perform the switching task between their dominant (L1) and a much less known language (L3). The critical difference is that the members of one group of participants are already high proficient bilinguals from birth (in a language not involved in the task) while the members of the other group are not. Note that for the sake of completeness we also compare early bilinguals controlling a dominant and a weak language (L1L3 EB group) vs. two dominant languages (Early bilinguals performing the same task in their two dominant languages; hereafter L1L2 EB). This second group comparison is not the main focus of the study and so it is presented subsequently.

In the ERP analysis, we pay special attention to the N2 and the Late Positive Component (LPC), two ERP components usually modulated in language control experiments. The N2 component has been associated with response selection and cognitive control processes (e.g., Kok, 1986; Van Boxtel et al., 2001; Nieuwenhuis et al., 2003; Donkers and Van Boxtel, 2004; Falkenstein, 2006; Gajewski et al., 2008; for a recent and extensive review see Folstein and Van Petten, 2008). In the linguistic domain, the amplitude of the N2 component appears to be affected by language control mechanisms during language switching and has been suggested to be indicative of the amount of inhibition (or, alternatively, conflict resolution) required to select an appropriate lexical item (Jackson et al., 2001; Christoffels et al., 2007; but see Verhoef et al., 2009). The LPC, at least in language, is thought to reflect the reconfiguration of stimulus-response mappings which is necessary to switch from naming a picture in one language to naming it in another language. That is, the LPC reflects the cognitive process of linking the input to the correct lexical item in the intended language (e.g., Liotti et al., 2000; Jackson et al., 2001). In other words, while the N2 seems more strongly associated with control in general, the LPC seems more strongly related to the consequences of this control at the level of specific lexical representations.

In sum, we test the following hypotheses: If early bilinguals indeed control their languages differently than late bilinguals, one could expect different N2 modulations in the L1L3 EB and L1L3 LB groups. Alternatively, if early bilinguals control a dominant and a third language in the same manner as late bilinguals do, no difference between L1L3 EB and L1L3 LB groups is expected. For the LPC, which does not reflect control *per se*, it can be expected that the L1L3 EB group would display a pattern similar to the L1L3 LB group since both groups have to make reconfigurations between a strong and a weak language.

## Materials and methods

### Participants

Twelve native speakers of Spanish, late learners of Catalan, formed the first group of the experiment (L1L3 LB group). They had English as a second language, but were tested in Catalan (L3) in order to compare the two groups of bilinguals when they control L1 and L3. They named pictures alternatively in Spanish (L1) and Catalan (L3). Twelve high proficient Spanish-Catalan early bilinguals, late learners of English, formed the second group of the experiment (L1L3 EB group). They named pictures alternatively in Spanish (L1) and English (L3). Finally, twelve high proficient Spanish-Catalan early bilinguals formed the third group of the experiment (L1L2 EB group). They named pictures alternatively in Spanish (L1) and Catalan (L2). Self-assessed proficiency ratings and language history are shown in Table 1,^3^. All participants had normal or corrected to normal vision and did not suffer neurological or motoric problems. For their participation in the experiment, participants received a course credit or monetary compensation.

**Table 1:** **Language history and self-assessed proficiency for the three groups of participants**.

	**Language history**			**Self-assessed proficiency**		
	**Age**	**L2 onset**	**L3 onset**	**L1**	**L2**	**L3**
L1L3 LB	24 (4)	10 (2)	22 (2)	4.0 (0.1)	2.1 (0.8)	2.2 (0.9)
L1L3 EB	21 (2)	2 (3)	10 (3)	4.0 (0.05)	3.8 (0.3)	2.2 (0.9)
L1L2 EB	21 (3)	4 (3)	9 (3)	4.0 (0.05)	3.9 (0.2)	2.2 (0.7)

*The “Language history” columns display, for each group of participants, (1) mean participants' age (in years); (2) the onset of L2 acquisition, which is the mean age (in years) at which EB participants started to learn Catalan and LB participants started to learn English; (3) the onset of L3 acquisition, which is the mean age (in years) at which EB participants started to learn English and LB participants started to learn Catalan. Standard deviations are reported into brackets*.

*Participants did not differ in age. L1L3 LB participants learned their L2 and L3 significantly later than L1L3 EB and L1L2 EB participants (all ps < 0.001)*.

*The “Self-assessed proficiency” columns display the proficiency scores obtained through a questionnaire fulfiled by the participants after the experiment. The scores are on a 4 point scale, in which 4 represents native speaker level; 3, good level; 2, medium level; and 1, bad level of proficiency. The self-assessed index reported is the average of the participants' responses to four domains (speech comprehension, speech production, reading, and writing)*.

*L1L3 LB proficiency in L1, L1L3 EB proficiencies in L1 and L2 and L1L2 EB proficiencies in L1 and L2 did not differ (all ps > 0.05). L1L3 LB proficiency in L2 significantly differed from L1L3 EB and L1L2 EB proficiencies in L2 (all ps < 0.001). Proficiencies in L3 did not differ between the three groups (all ps > 0.9)*.

### Task and procedure

For the first two groups, ten pictures of common objects with non-cognate names and from various semantic categories were used as experimental stimuli (the same ones used by Costa and Santesteban, 2004, Experiment 1A). The Catalan and Spanish names of the pictures were matched for length (5.7 vs. 5.6 phonemes, respectively; see **Appendix A**). The Spanish-English translations were non-cognate words (length in phonemes: 6.1 vs. 5.6). The materials slightly differed for the third (L1L2 EB) group. Because the current experiments meant to mimic those of Costa and Santesteban (2004) and in order to increase power, we increased the number of pictures to 40 (the ones used in Experiment 3 of Costa and Santesteban, 2004; see **Appendix A**)^4^. All pictures had non-cognate names and were from various semantic categories. Spanish and Catalan words were matched for length (5.1 and 5.2 phonemes, respectively). The two lists of 40 and 10 words did not differ in terms of mean word frequency, number of letters, number of orthographic and phonological neighborhoods, number of phonemes and syllables, familiarity, concreteness, and imageability (all *p*s > 0.11).

The language in which participants had to respond was determined by the color in which the picture appeared (blue: naming in Catalan/English; red: naming in Spanish, or conversely). The color-language association was counterbalanced across participants, but all pictures appeared in both languages. There were four types of trials randomly presented: no switch in L1, no switch in L2 (or L3), switch into L1 and switch into L2 (or L3).

Although recording of EEG in an immediate overt naming task provides reliable brain responses (e.g., Christoffels et al., 2007; Costa et al., 2009b; Verhoef et al., 2009; Strijkers et al., 2010, 2011), we chose here to adopt the same delayed naming strategy as employed in Jackson et al. (2001). The main reason for doing so was that we were interested in exploring ERP components in a large time-window. For instance, we were interested in group effects on the LPC, a component with a time-frame that closely overlaps with immediate speech onset. The two immediate naming ERP studies on language switching in the literature so far have failed to observe the LPC (Christoffels et al., 2007; Verhoef et al., 2009). We therefore, opted for the following procedure: We asked participants to provide a verbal response as fast as possible but not before the target stimulus disappeared from the screen. Crucially, pictures could disappear either 250 ms or 1000 ms after onset. These two types of trials were randomly presented, making it unpredictable whether a given trial would require an immediate response (250 ms trials) or a delayed one (1000 ms trials). Thus, participants had to start processing the target as soon as it was presented, even when the delay was 1000 ms. This design allowed us to (a) use the trials of the 250 ms delay to assess the pattern of naming latencies, and (b) use the trials of the 1000 ms delay to analyze the ERPs.

Each participant was presented with 950 trials (70% no-switch and 30% switch; each picture was presented 95 times during the experiment). Half of the no switch trials had to be named in L1 and half in L2 (or L3). The same applied for the switch trials. Therefore, participants used their L1 and L2 (or L3) the same number of times overall (475 responses in each language). Given the increase in the number of pictures for the L1L2 EB group, there was a decrease in the number of times a given picture was presented: 23 or 24 times in total.

Participants were tested individually in a soundproof room. They were instructed to name the pictures as fast as possible, but only after they had disappeared from the screen. Each list of trials started with the presentation of a red or blue circle with the word *Español* (Spanish) or *Català* (Catalan) written below for 2000 ms (words *Español* and *English* were displayed for the L1L3 EB group). The circle indicated the language in which the first picture of a list had to be named. After 2000 ms the first picture appeared on the screen and remained for 250 ms or 1000 ms. The language used to name the picture was determined by the color of the picture. After the participant's response (at stimulus offset) or for a maximum response limit of 2000 ms, a blank interval of 1150 ms was presented, and the next trial started. Naming latencies were measured from stimulus offset until response onset. Before the actual experiment started, participants were familiarized with the name of the pictures in both languages and familiarized with the task by a training phase.

Error rates and naming latencies were submitted to analyses of variance (ANOVAs) with Group (L1L3 LB vs. L1L3 EB or L1L3 EB vs. L1L2 EB), Type of Trial (Switch vs. No-switch), and Language of response [L1 vs. L2 (or L3)] as factors.

### Electrophysiological recording and analyses

Electrophysiological data were recorded (Brain Vision Recorder 1.05; Brain Products) in reference to an electrode placed on the participant's nose at a rate of 250 Hz from 31 tin electrodes placed according to the 10–20 convention (FPz, FP1, FP2, Fz, F3, F4, F7, F8, FC1, FC2, FC5, FC6, Cz, C3, C4, T3, T4, CP1, CP2, CP5, CP6, Pz, P3, P4, T5, T6, PO1, PO2, Oz, O1, O2). Impedances were kept below 3 kΩ. EEG activity was filtered off-line with a 30 Hz low-pass filter and a 0.03 Hz high-pass filter (24 dB). Eye blink artifacts were mathematically corrected using Gratton and Coles (1989) procedure, implemented in Brain Vision Analyzer 1.05 (Brain Products), and any remaining artifacts were manually dismissed. Trials where the participant's response was incorrect, absent, or before stimulus offset, were rejected from the dataset before averaging. Epochs ranged from −100 to 1000 ms after the stimulus onset. Baseline correction was performed in reference to pre-stimulus activity and individual averages were digitally re-referenced to a global reference. For each condition, the grand average was obtained by averaging individual averages. ERP components were defined based on the mean global field power measured across the scalp, which summarizes the contribution of all electrodes in the form of a single vector norm (Picton et al., 2000). This procedure was applied on the grand averages obtained for each condition. For each component observed on grand averages, the electrode of maximal amplitude of the peak was defined as the “referent electrode.” The “reference latency” of each peak corresponded to the latency over the referent electrode. The interval of each component was the time-window centered on the “reference latency” value and with a duration based on visual inspection of the mean global field power. This allowed automatic peak detection in the following intervals (for each individual average): 250–350 ms for the N2 and 500–650 ms for the LPC^5^. Individual mean amplitudes (average of the ERP amplitude in a given interval) were measured for each component and each participant. Latencies were detected on individual averages, for each component and each participant (except for the LPC, because individual latency values could not be detected in each participant).

Peak latencies and mean amplitudes were submitted to a repeated measures ANOVA with Group (L1L3 LB vs. L1L3 EB or L1L3 EB vs. L1L2 EB), Type of Trial (Switch vs. No-switch), Language of response [L1 vs. L2 (or L3)], Hemisphere (Left vs. Right) and Electrode (2 sites) as factors. Each component was studied at the four electrode sites with maximal mean peak amplitude. The N2 component was studied over frontal sites (F3, FC1, F4, FC2) and the LPC was studied over parietal sites (C3, CP1, C4, CP2).

## Results

### Behavioral results

Analysis of naming latencies is based on the trials in the 250 ms delay condition. We will first compare the L1L3 LB and the L1L3 EB groups to answer the main question on the effect of early bilingual control mechanism. Next, we will explore whether early bilingual language control depends on the strength of the languages at play by comparing the L1L3 EB and the L1L2 EB groups. We present the results in this way for the sake of clarity, but note that a global ANOVA comparing all three groups led to identical results to the ones presented below (see **Appendix B** for detailed results of the general ANOVA).

Two types of responses were scored as errors: (a) verbal disfluencies (stuttering, utterance repairs, production of non-verbal sounds that triggered the voice key); (b) trials in which participants produced a different name from that designated by the experimenter. Responses exceeding 3 standard deviations from the participant's mean were also removed. Following these criteria, 5% of the trials were excluded from the analyses in the L1L3 EB group and 2.2% in the L1L3 LB group.

In the error analysis, the only significant effect was the group effect [*F*_(1, 22)_ = 6.38; *p* = 0.02] showing that the L1L3 EB group produced significantly more errors than the L1L3 LB group. There were no other main effects or interactions (all *p*s > 0.3).

**Figure 1:**
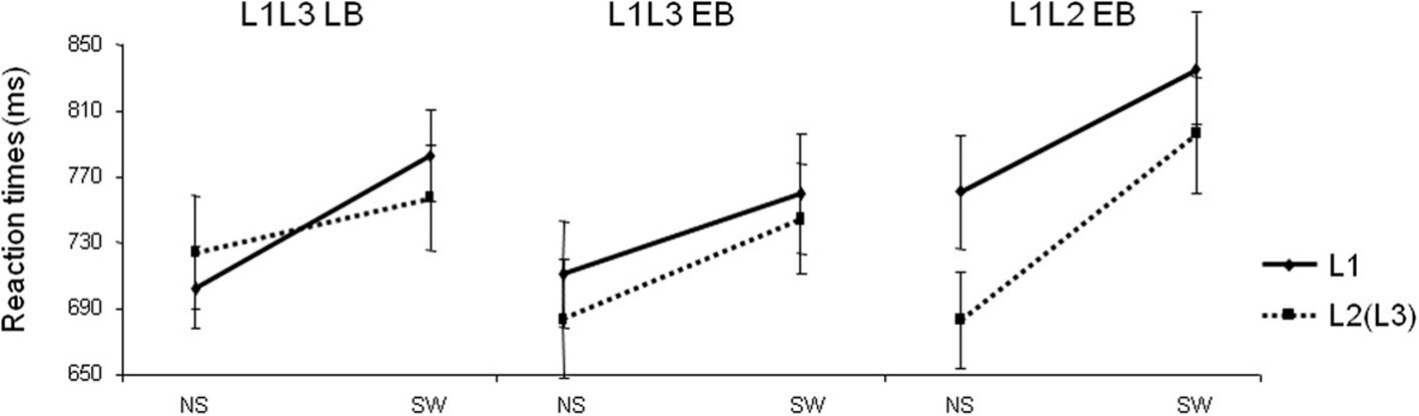
**Mean naming latencies (reaction times in milliseconds) of Late L3 learners (L1L3 LB), Early bilingual L3 learners (L1L3 EB), and Early bilinguals switching between their two dominant languages (L1L2 EB) in the four conditions of the task.** L1, response given in L1; L2(L3), response given in L2 or L3; NS, no switch trial; SW, switch trial. Error bars represent standard errors.

The naming latency analysis did not reveal any group effect [*F*_(1, 22)_ < 1; *p* = 0.70], showing that both groups of participants were comparable in terms of global naming latencies (L1L3 EB group: 725 ± 121 ms; L1L3 LB group: 742 ± 105 ms). There was a main effect of Type of Trial [*F*_(1, 22)_ = 159.69; *p* < 0.001] revealing a general switch cost, meaning that switch trials were named slower than no switch trials (see Figure 1). The Group × Type of Trial × Language interaction was significant [*F*_(1, 22)_ = 6.21; *p* = 0.02] and there were no other significant main effects or interactions (all *p*s > 0.1). *Post-hoc* analysis of the triple interaction (Scheffé test) revealed that there was a significant switch cost in L1 (*p* = 0.04) and in L3 (*p* = 0.007) in the L1L3 EB group. In other words, switch trials were named slower than no switch trials both in L1 and L3. For the L1L3 LB group, the switch cost was significant only in L1 (*p* = 0.0003). Separate ANOVAs for each group of participants revealed a significant Type of Trial × Language interaction in the L1L3 LB group [*F*_(1, 11)_ = 6.10; *p* = 0.03] but not in the L1L3 EB group [*F*_(1, 11)_ = 0.69; *p* = 0.42]. Summarizing, the L1L3 LB and L1L3 EB groups did not have the same behavioral pattern of language switching; the pattern was asymmetrical in late and symmetrical in early bilinguals. These results are in line with Costa and colleagues (Costa and Santesteban, 2004; Costa et al., 2006).

Behavioral results for the L1L2 EB group were analyzed and compared to the L1L3 EB group. Removing errors and responses exceeding 3 standard deviations from the participant's mean, 3.4% of the trials were excluded from the analyses in the L1L2 EB group.

In the error analysis, no main effect or interaction was significant (all *p*s > 0.12). The naming latency analysis did not reveal any group effect [*F*_(1, 22)_ = 0.87; *p* = 0.36], showing that participants of the two groups were comparable in terms of global naming latencies (L1L3 EB group: 725 ± 121 ms; L1L2 EB group: 768 ± 125 ms; Figure 1). There was a main effect of Type of Trial [switch trials slower than no switch; *F*_(1, 22)_ = 125.42; *p* < 0.001], a Language of response effect [responses in L1 slower than in L2/L3 in both groups; *F*_(1, 22)_ = 19.28; *p* < 0.001] and a Group × Type of Trial interaction [*F*_(1, 22)_ = 7.66; *p* = 0.01]. There were no other main effects or interactions (all *p*s > 0.05). *Post-hoc* analysis of the Group × Type of Trial interaction (Scheffé test) revealed that the switch cost was significant in both L1L2 EB (*p* < 0.001) and L1L3 EB groups (*p* < 0.001). The main pattern of behavioral switch cost did not significantly differ in the two groups of early bilinguals.

### Event-related potential (ERP) results

**Figure 2:**
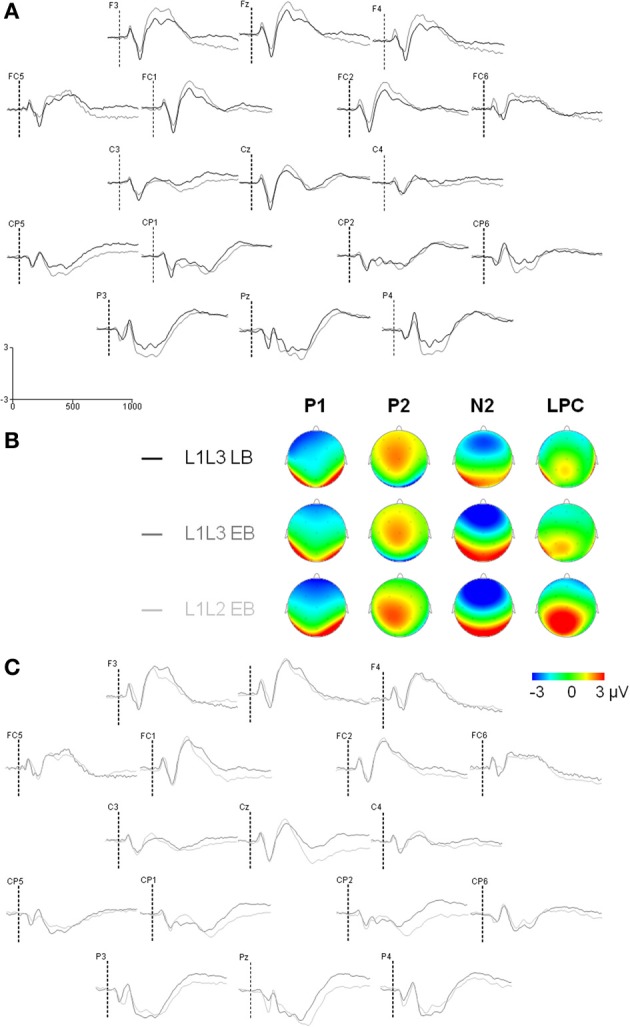
**Event-related potentials (Y axis: Mean amplitude in μ V) from the trial presentation (time 0) to 1000 ms. (A)** ERPs elicited by the presentation of a picture, for L1L3 LB group (black waves) and L1L3 EB group (dark gray waves) over the scalp (17 electrode sites). **(B)** Topographic maps for the four main ERP components (P1: 90 ms, P2: 160 ms, N2: 290 ms, LPC: 490 ms) for L1L3 LB, L1L3 EB and L1L2 EB groups. Color scale ranges from −3 to 3 μ V, except for P1 component for which it ranges from −2 to 2 μ V. **(C)** ERPs elicited by the presentation of a picture, for L1L3 EB group (dark gray waves) and L1L2 EB group (light gray waves) over the scalp (17 electrode sites).

The ERP analyses are based on the 1000 ms delay condition. Trials in which participants made naming errors, or that led to recording artifacts were removed from the analyses (L1L3 EB group: 5.4% switch, 4.8% no-switch; L1L3 LB group: 2.3% switch, 2.1% no-switch). Except for the effect of hemisphere, peak latency analyses on the occipital P1, the frontal P2, and the frontal N2 did not reveal any significant main effect or interaction (As the LPC peak was not clearly observable in individual data, LPC latency was not analyzed). Mean amplitude analyses on the occipital P1 and the frontal P2 did not reveal any significant main effect or interaction (Figure 2). Thus, for the sake of clarity, only the analyses of the N2 and LPC mean amplitudes will be reported in the following section.

**Figure 3:**
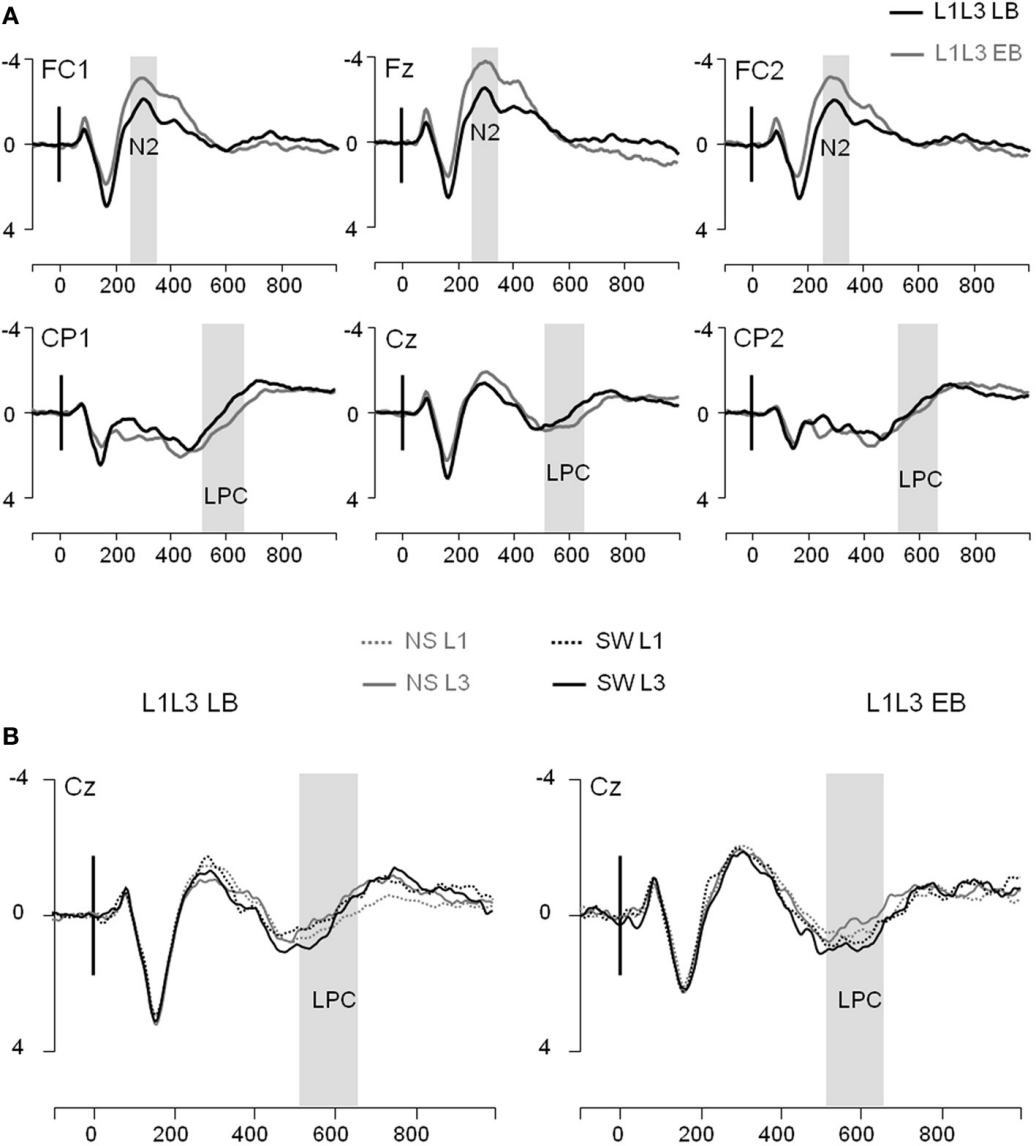
**Event-related potentials (Y axis: Mean amplitude in μV) from the trial presentation (time 0) to 1000 ms. (A)** ERPs elicited by the presentation of a picture, for L1L3 LB group (black waves) and L1L3 EB group (dark gray waves) at 6 midline electrodes (FC1, Fz, FC2, CP1, Cz, CP2). **(B)** ERPs elicited by a no switch in L1 trial (NS L1; dotted gray), a no switch in L3 trial (NS L3; solid gray), a switch into L1 trial (SW L1; dotted black) and a switch into L3 trial (SW L3; solid black). ERPs displayed for the L1L3 LB group (left panel) and the L1L3 EB group (right panel) at the central electrode Cz.

In the frontal region, there was a main Group effect on the N2 [*F*_(1, 22)_ = 6.59; *p* = 0.02] with no other significant main effect or interaction (all *p*s > 0.16). The N2 component was significantly larger in the L1L3 EB than in the L1L3 LB group (see Figure 3A)^6^. With regard to the LPC, in the parietal region, there was a significant Hemisphere effect [LPC larger on the left hemisphere; *F*_(1, 22)_ = 11.02; *p* = 0.003] and a significant Type of Trial × Language of response interaction [*F*_(1, 22)_ = 6.79; *p* = 0.02]. *Post-hoc* analysis revealed that the LPC mean amplitude was larger in switch than in no switch trials when responses were given in L3 (*p* = 0.002) but not when they were given in L1 (*p* = 1.00; Figure 3B). There was no Group effect [*F*_(1, 22)_ = 1.04; *p* = 0.32] nor any other main effect or interaction (all *p*s > 0.06).

The L1L3 EB ERP pattern was then compared to the ERP pattern of the L1L2 EB group (error or artifactual trials removed from ERP analyses: 3.8% switch, 3.3% no-switch).

**Figure 4:**
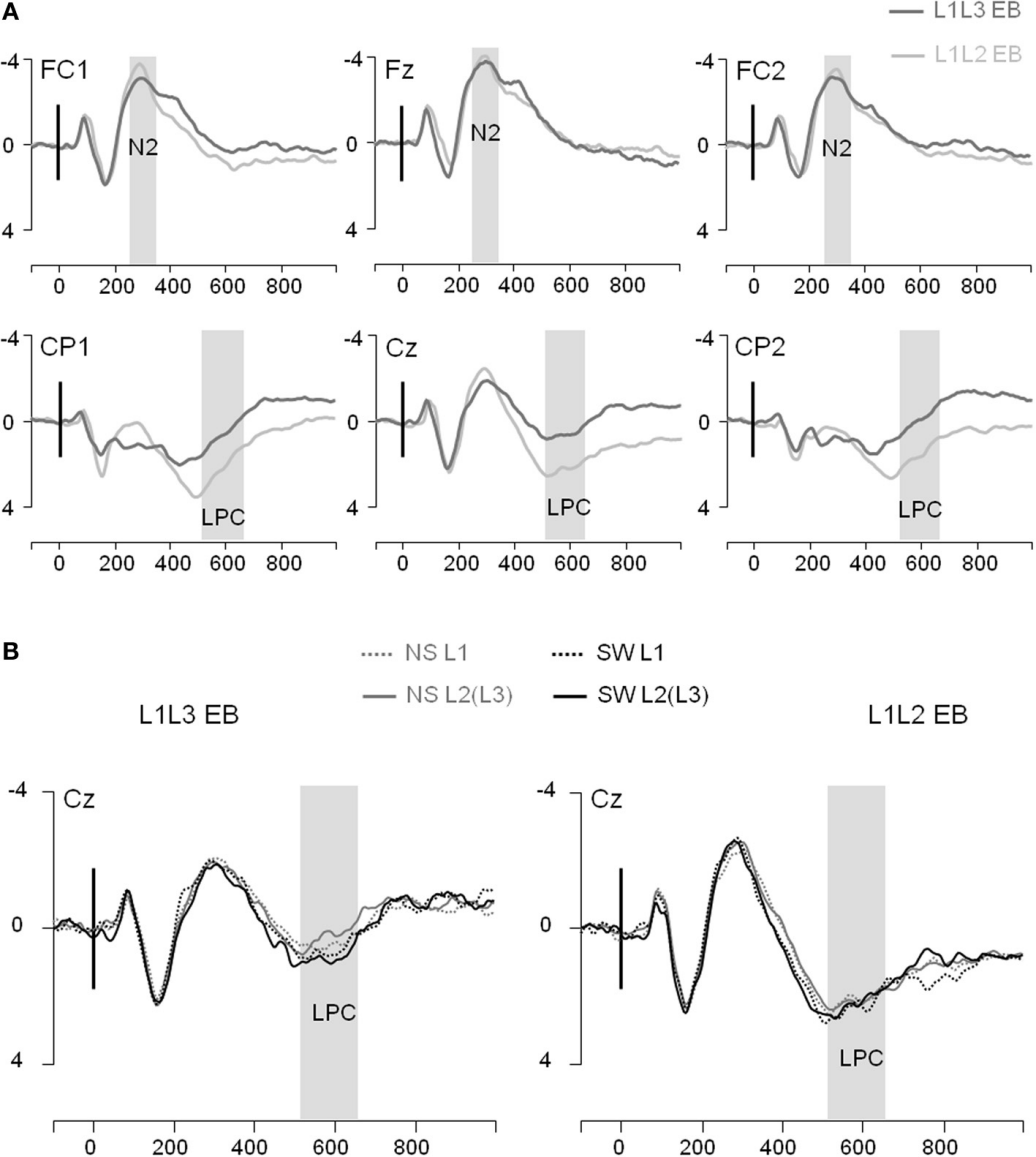
**Event-related potentials (Y axis: Mean amplitude in μ V) from the trial presentation (time 0) to 1000 ms. (A)** ERPs elicited by the presentation of a picture, for L1L3 EB group (dark gray waves) and L1L2 EB group (light gray waves) at 6 midline electrodes (FC1, Fz, FC2, CP1, Cz, CP2). **(B)** ERPs elicited by a no switch in L1 trial (NS L1; dotted gray), a no switch in L2(L3) trial [NS L2(L3); solid gray], a switch into L1 trial (SW L1; dotted black) and a switch into L2(L3) trial [SW L2(L3); solid black]. ERPs displayed for the L1L3 EB group (left panel) and the L1L2 EB group (right panel) at the central electrode Cz.

In the frontal region, the general ANOVA did not reveal any significant main effect or interaction on the N2 (all *p*s > 0.10; see Figure 4A). Regarding the LPC, the general ANOVA performed in the parietal region revealed a significant Group effect [*F*_(1, 22)_ = 4.02; *p* = 0.04]: The LPC was significantly larger in L1L2 EB than L1L3 EB participants. There was a significant Type of Trial effect [*F*_(1, 22)_ = 7.31; *p* = 0.01] and a significant Type of Trial × Language interaction [*F*_(1, 22)_ = 4.76; *p* = 0.04]. *Post-hoc* analysis revealed that the LPC mean amplitude was larger in switch than in no switch trials when responses were given in L2/L3 (*p* = 0.006) but not when they were given in L1 (*p* = 1.00; Figure 4B). The LPC was also larger on the left than on the right hemisphere [*F*_(1, 22)_ = 11.83; *p* = 0.002]. There were no other main effects or interactions (all *p*s > 0.13).

To summarize, the main observation relevant for our question was that the N2 ERP component was larger in the L1L3 EB than in the L1L3 LB group. In this time-window, both groups of early bilinguals (L1L3 EB and L1L2 EB) did not differ. The LPC did not differ between the two groups tested in a dominant and a weak language (L1L3 EB and L1L3 LB), but this component was larger in L1L2 EB than L1L3 EB participants.

## Discussion

The main goal of our study was to explore whether early and late bilinguals control a dominant and a late acquired language (L3) in the same manner. To answer this question, we explored the electrophysiological correlates of language control in two groups of participants: late L3 learners who did not learn two languages from birth (L1L3 LB) and early bilinguals learners of an L3 (L1L3 EB).

### ERP pattern of language control in early and late bilinguals

Our most important novel finding is the difference in brain activity between early and late bilinguals (L1L3 EB vs. L1L3 LB groups) when handling a dominant and a weak language. We focused our investigation on the N2 and LPC, two ERP components usually encountered in language control experiments.

Around 300 ms after picture onset, we observed that the anterior N2 component was larger for late bilinguals than for early bilinguals. The functional interpretation of the anterior N2 in cognitive control tasks is debated. Some studies suggest that it reflects inhibitory processes (e.g., Kok, 1986; Falkenstein et al., 1999; Jackson et al., 1999; Van Boxtel et al., 2001), while others suggest that the anterior N2 is involved in conflict monitoring (e.g., Nieuwenhuis et al., 2003; Donkers and Van Boxtel, 2004). Regardless of the specific cognitive processes revealed by the N2, what is important for the present purposes is that such a component seems to be somehow involved in response selection processes and cognitive control (e.g., Kok, 1986; Van Boxtel et al., 2001; Nieuwenhuis et al., 2003; Donkers and Van Boxtel, 2004; Falkenstein, 2006; Gajewski et al., 2008; for an extensive review see Folstein and Van Petten, 2008). In this context, our observations suggest that the linguistic status (the fact of being a high proficient bilingual from birth or not) has a significant influence on cognitive control processes. At the very least, this observation reveals that early bilingual learners of a third language do not control this new language as late bilingual L3 learners do.

Regarding later components, the LPC was not modulated by the linguistic status of participants. The LPC is usually interpreted as reflecting the reconfiguration of stimulus-response mapping necessary to make a language switch (e.g., Konishi et al., 1998; Liotti et al., 2000; Wylie et al., 2003). Thus, the LPC is probably more related to the consequences of language control at the level of specific lexical representations; that is, it is sensitive to the selection of the lexical item in the intended language. If so, we can conclude that as soon as early and late bilinguals have to control languages comparable in strength (a dominant and a weak language), they do not differ in terms of stimulus-response mapping reconfiguration.

One potential caveat when interpreting these data is the possibility that the two groups of bilinguals differed in how well they knew the words used in the task. We believe that this explanation is unlikely for the following reasons: First, the use of a small set of words (only 10 different pictures, corresponding to 10 high frequency words) makes it unlikely that differences in learning vocabulary would have an important impact in the task (cf. **Appendix A**); Second, the performance of the two groups in terms of accuracy and speed were similar (if anything, the L1L3 EB group had lower levels of accuracy than the L1L3 LB group); Third, if anything, the L1L3 LB group should be the one to benefit from better vocabulary size: It is easier for Spanish speakers living in Catalunya to acquire vocabulary in Catalan than in English (given the similarity between Spanish and Catalan and the immersion environment). Then, in our view, the fact that L3 differed between the two groups (Catalan for late bilinguals and English for early bilinguals) cannot explain the main group difference observed in language control. Note also that L1L2 EB participants were doing the task in Catalan, and their pattern of results mimics the one of L1L3 EB (using English) and not the one of L1L3 LB (also using Catalan). Nevertheless, further research should be conducted with early and late bilinguals tested in the same languages.

Another potential caveat is the fact that L1L3 EB participants acquired L3 earlier than L1L3 LB participants (cf. Table 1). It is unlikely because language control does not seem to depend on the age of acquisition of the weak language (Costa et al., 2006). Moreover, L1L3 LB bilinguals acquired their L3 late in life, but they were using it on a daily basis at the time of testing (Spanish natives living in Catalunya and learning Catalan for 1 year). On the contrary, participants of the three groups never used English on a daily basis since they used it only at school. Thus, L1L3 LB had more opportunities to develop cognitive control mechanisms in handling L1 (Spanish) and L3 (Catalan) than any participant of any of the three groups had in handling L1 (Spanish) and English. The main caveat in the difference in age of acquisition comes from the fact that L1L3 EB participants acquired L3 during the critical period of puberty (see extensive literature on critical period in language acquisition; e.g., Mayberry and Eichen, 1991; Weber-Fox and Neville, 1996; Perani et al., 1998; Pallier, 2007). Learning a second language earlier or later than age 10 is known to have important consequences on how semantic and syntactic information is processed in L2 (Weber-Fox and Neville, 1996). Nevertheless, we are confident that naming 10 pictures of high frequency should not be as strongly affected by age of acquisition as sentence comprehension. In any case, further research needs to be conducted to assess the impact of age of acquisition on the language control system, and the relevance of the critical period for such cognitive capacity.

Finally, we have to acknowledge that the L1L3 EB group and the L1L3 LB group do not only differ in their linguistic status (being early vs. late bilinguals) but also in L2 proficiency. Participants being tested in L1 and L3, proficiency in L2 should not directly affect cognitive capacities for the present task. Nevertheless, we have to consider two alternative interpretations for the current data: Differences in language control between L1L3 EB and L1L3 LB participants might be due to the fact that the former acquired two languages from birth, as we argued so far. On the other hand, L1L3 EB participants might control languages in a different manner because they achieved native-like proficiency in another language than L1, which was not the case for L1L3 LB participants. Testing early bilinguals with low proficiency in L2 or late bilinguals having achieved native-like proficiency in L2 is difficult. Nevertheless, such groups of participants should be tested in the future in order to tease apart the contribution of native-like proficiency and/or early age of acquisition in language control. At the very least, we can conclude that early bilingual learners of an L3 do not control languages in the same way as late bilingual L3 learners –who have not achieved native-like proficiency in their L2– do.

### ERP pattern of language control in early bilinguals using languages of different relative strength

The comparison of the two groups of early bilinguals tested in their two dominant languages (L1L2 EB) or in a dominant and a weak language (L1L3 EB) gives us the opportunity to make a first approximation of how the language control system developed by early bilinguals behaves as a function of dominance of the two languages involved in the task. It is particularly interesting to assess the differences between these two groups in the time-window where differences between late and early bilinguals were observed (i.e., the N2 component). Whereas this component was sensitive to the linguistic status of the participants (early vs. late bilinguals), it did not show any sensitivity to the relative strength of the languages involved in the switching task. That is, early bilinguals showed the same N2 amplitude regardless of whether the task involved their two dominant languages (L1L2 EB) or one of their strong languages and a weak one (L1L3 EB). This observation is another argument in favor of the assumption that early and late bilinguals differ in the way they control languages. The similar N2 pattern in these two groups of participants, who were using Spanish/English vs. Spanish/Catalan as the task languages, also suggests that the nature of the weak language and its proximity to the dominant language is not a major factor influencing language control.

Regarding stimulus-response mapping reconfiguration, we concluded based on the results of the L1L3 LB and L1L3 EB comparison that this process is independent of the linguistic status of bilinguals and might rather depend on the relative strength of both languages at play. If this is true, the LPC should be different in the L1L2 EB and the L1L3 EB group, as the former had to control two dominant languages and the latter had to control a dominant and a weak language. Indeed, we observed that the LPC was significantly larger in L1L2 EB than in L1L3 EB participants, which confirms our previous assumption.

However, one should be cautious when drawing strong conclusions because of the differences in the number of pictures used in both groups of participants. Further research is needed to establish whether the LPC mean amplitude is influenced by the languages involved and/or by the number of pictures.

### Behavioral switch costs in the three groups of participants

The behavioral results of the L1L3 LB and L1L3 EB groups replicated previous findings (Meuter and Allport, 1999; Jackson et al., 2001; Costa and Santesteban, 2004; Costa et al., 2006; but see Christoffels et al., 2007; Gollan and Ferreira, 2009; Verhoef et al., 2009 for divergent results). First, L1L3 LB participants showed an asymmetrical switch cost: Switching from L3 to L1 was more costly than switching from L1 to L3. Second, L1L3 EB participants showed symmetrical switch costs: Switching from one language to the other was as costly as the other way around. Note that the symmetrical switch cost was also observed when early bilinguals switched between their two dominant languages (L1 and L2). To the extent that one can consider switching data as actually informative for natural language control, this pattern of results suggests that early and late bilinguals control their lexicalization process during speech production in a different manner.

We also observed a significant “paradoxical language effect” in both early bilingual groups. In these two groups, naming latencies were slower in the dominant language (L1) than in the other two languages (L2 and L3). This is an interesting effect that has been previously observed in several language switching experiments (see Costa and Santesteban, 2004; Costa et al., 2006; Christoffels et al., 2007; Kroll et al., 2008 and Verhoef et al., 2009 for a review). One tentative functional explanation for this “paradoxical language effect” is that, during language switching tasks, early and high proficient bilinguals are able to raise the activation of the lexical representations of the non-dominant language, making them more available (Costa and Santesteban, 2004). It would lead them to suffer less from language switching, but consequently, to be slower in naming in their dominant language. On the contrary, slower naming latencies in L1 can be explained by a reduced availability of lexical representations in L1. This control would be possible by globally inhibiting the L1 or by selectively increasing its activation threshold (Christoffels et al., 2007; see also Kroll et al., 2006, 2008).

The interesting observation for our purposes is that the slowing down of naming latencies in L1 was observed in early but not in late bilinguals. Thus, whatever the functional explanation of the effect, it is another argument in favor of a different way in controlling languages in early and late bilinguals. Nevertheless, we should be cautious in drawing strong conclusions on the “paradoxical language effect” as it has been observed previously in late bilinguals (see for instance Costa and Santesteban, 2004).

### ERP pattern of the switch cost

Looking at the pattern of the N2 component, we did not observe any clear signature of the switch cost (no significant effect of Type of Trial or Language of response). This lack of a clear language switch signature adds to the concern raised by other studies about the systematicity of the ERP results in relation to language switch costs (Jackson et al., 2001; Christoffels et al., 2007; Verhoef et al., 2009). Nevertheless, it seems that the predictability of the switch has a major impact on the N2 component: When the switch of Language of response can be predicted, the N2 component is affected by the Type of Trials (switch or no switch). This was observed in paradigms with regular alternations of switch and no switch trials (Jackson et al., 2001), and in paradigms with the language cue displayed before the trial and not simultaneously (Verhoef et al., 2009). On the contrary, in this experiment as in a previous one (Christoffels et al., 2007), switch and no switch trials were unpredictable, and the N2 component was not affected by the switch cost. All these findings together suggest that the N2 component in language switching might be influenced more by preparatory processes than by the language switch *per se*.

Later on during language production, the LPC seems to be an ERP component more clearly associated to language switching. As stated above, the LPC is interpreted as reflecting stimulus-response mapping reconfiguration (e.g., Konishi et al., 1998; Liotti et al., 2000; Wylie et al., 2003). In our study, the LPC was larger for switch than no switch trials for weak languages (L3 for L1L3 LB group and L1L3 EB group). It was not modulated by the Type of Trial when the language in use was dominant (L1 for the three groups and L2 for L1L2 EB). These results are in accordance with the hypothesis previously formulated that the LPC (as encountered in language switching studies) is sensitive to the selection of the lexical item in the intended language, as well as to the strength of the language at play. As stated earlier in the discussion, we have to be cautious in drawing strong conclusions on the LPC effects. Nevertheless, our results are consistent with previous research in showing that the more complex the processing of the stimulus (switch vs. non-switch condition; weak vs. dominant language), the more cognitively demanding the stimulus-response mapping reconfiguration (larger LPC mean amplitude; Jackson et al., 2001; Moreno et al., 2002; Khateb et al., 2007; Kieffaber et al., 2013). More importantly for our purpose, we can conclude that stimulus-response mapping reconfiguration is not the locus of the difference in the way early and late bilinguals control languages. This cognitive process is modulated by the complexity of the cognitive control, apparently in the same manner in early and late bilinguals. Nevertheless, future investigations are needed to interpret the exact influence of the Type of Trial and the Language of response on this component, as results from different studies are inconsistent. For example, Jackson et al. (2001) observed a larger LPC for switch than no switch trials, both for L1 and L2, and the LPC was not influenced by the Type of Trial in Christoffels et al.'s study (2007).

## Conclusion

The main goal of our study was to investigate whether or not having reached proficiency in a second language early in life affects control mechanisms applied on a dominant and a late acquired language. We observed that early bilingual learners of an L3 do not control languages in the same way as late bilingual L3 learners –who have not achieved native-like proficiency in their L2– do. Even if the relative strength of the languages is similar in both groups, they control their language output in a different manner. Such difference in language control could, at least in part, be responsible for the bilingual advantage in learning a new language. We can conclude from the present data that the two groups do not control languages in the same manner. Nevertheless, we cannot argue whether this difference is qualitative or quantitative. We cannot either strongly conclude on the locus of the difference, even if we can tentatively argue that the locus of the difference in the way early and late bilinguals control languages is in preparatory processes. The group difference arguably does not stem in stimulus-response mapping reconfiguration. Further extensive research is needed to define precisely why and how early bilingual learners of an L3 do not control languages in the same way as late bilingual L3 learners do.

This study proposes a novel way to compare language control in different populations of bilingual speakers. We suggest that main differences in controlling languages are due to the fact of being early bilingual or not. Thus, early and late bilinguals should differ in the way they control a dominant and a weak language, whatever the age of acquisition and the proficiency of the weak language (as long as it was not acquired from birth). Our study is the first step toward that conclusion. In the future, studies should be conducted to understand the precise nature of the control mechanisms and how they are affected by the task (using another task than the language switching paradigm) and the languages (testing languages with different ages of acquisition and proficiencies).

### Conflict of interest statement

The authors declare that the research was conducted in the absence of any commercial or financial relationships that could be construed as a potential conflict of interest.

## Footnotes

^1^Both groups are tested in a late acquired third language and have the same proficiency in that language (cf. Table 1 but the L3 is English for early bilinguals and Catalan for late bilinguals (different ages of acquisition). Another option would be to test both groups in English (same age of acquisition). Nevertheless, English is the second language of late bilinguals and the third language of early bilinguals. We choose here to compare the two groups in their third language to avoid the confounding effect of comparing groups handling an L2 vs. L3.

^2^Note that many studies in the field also compare high vs. low proficient bilinguals, and that age of acquisition and proficiency are even sometimes confounded (comparison of early/high proficient and late/low proficient bilinguals). In the present study, we will focus on the comparison between early and late bilinguals.

^3^Even if these are not objective measures, we are quite confident in self-rating values, as it has been shown that subjective measures of self-rating language proficiency provide an effective measure of bilingual ability (cf. Marian et al., 2007). Moreover, self-assessment is commonly used in studies comparing groups of bilinguals with equivalent or different language proficiency (see for instance Costa and Santesteban, 2004).

^4^Although one may argue that this manipulation decreases comparability between experiments, this difference was not supposed to qualitatively influence language control mechanisms, as previous research has shown that an increase in the number of pictures does not affect the pattern of naming latencies and language switch cost (Costa and Santesteban, 2004). In any case, any similarity between the two early bilingual groups (especially in terms of ERP pattern) will be even more convincing given this different amount of stimuli in the two tasks.

^5^Visual inspection of the data also suggested the presence of two other ERP components: An occipital P1 in the [80–120] ms time-window and a frontal P2 in the [150–180] ms time-window. Analyses of variance performed on mean amplitudes and latencies of these components did not reveal any significant main effect or interaction, except for the effect of hemisphere.

^6^To make sure that the main group effect observed on the N2 mean amplitude was not driven, at least partly, by a tendency for a group effect on the P2 component, we performed an ANOVA on peak-to-peak amplitudes (amplitude of the P2 minus amplitude of the N2). This analysis confirmed that the N2 component was significantly larger in the L1L3 EB than in the L1L3 LB group.

## Appendix A: materials used in the experiment

The whole set of words was used in L1L2 EB group. In L1L3 LB and L1L3 EB groups, only the 10 words marked with an asterisk were used.

**Table:**

**Spanish name**	**Catalan name**	**English name**
^*^Sombrero	Barret	Hat
^*^Zanahoria	Pastanaga	Carrot
^*^Manzana	Poma	Apple
^*^Mesa	Taula	Table
^*^Perro	Gos	Dog
^*^Hoja	Fulla	Leaf
^*^Cuchillo	Ganivet	Knife
^*^Ventana	Finestra	Window
^*^Lluvia	Pluja	Rain
^*^Queso	Formatge	Cheese
Rama	Branca	Branch
Gusano	Cuc	Worm
Red	Xarxa	Net
Jaula	Gabia	Cage
Corcho	Suro	Cork
Jamon	Pernil	Ham
Melocoton	Pressec	Peach
Mujer	Dona	Woman
Hueso	Os	Bone
Dedo	Dit	Finger
Huevo	Ou	Egg
Ojo	Ull	Eye
Pañuelo	Mocador	Handkerchief
Muleta	Crossa	Crutch
Calcetin	Mitjo	Sock
Rana	Granota	Frog
Pimiento	Pebrot	Pepper
Vela	Espelma	Candle
Buho	Mussol	Owl
Cepillo	Raspall	Brush
Cerdo	Porc	Pig
Silbato	Xiulet	Whistle
Pato	Anec	Duck
Mariposa	Papallona	Butterfly
Silla	Cadira	Chair
Mancha	Taca	Blot
Burro	Ase	Donkey
Hoz	Falc	Sickle
Zueco	Esclop	Clog
Colchon	Matalas	Mattress

## Appendix B: results of the main ANOVA on error rates, naming latencies and ERPs including the three groups of participants

Behavioral data were analyzed using three factors: Group (L1L2 EB vs. L1L3 EB vs. L1L3 LB), Type of Trial (Switch vs. No-switch), and Language of Response (L1 vs. L2/L3).

**Table:**

	*F*-value	*p*-value
**GENERAL ANOVA ON ERROR RATES**		
*Group*	*3.88*	*0.03*^a^
Type of trial	1.96	0.17
Language of response	2.10	0.16
Group × type of trial	0.08	0.92
Group × language of response	0.63	0.54
Type of trial × language of response	0.29	0.60
Group × type of trial × language	0.79	0.46

a *L1L3 LB made fewer errors than L1L3 EB. The italic values are indicates that p < 0.05*.

**Table:**

	*F*-value	*p*-value
**GENERAL ANOVA ON NAMING LATENCIES**		
Group	0.50	0.61
*Type of trial*	*179.38*	*<0.001*
*Language of response*	*8.06*	*0.008*
*Group × type of trial*	*5.71*	*0.007*
Group × language of response	2.97	0.07
Type of trial × language of response	0.001	0.98
*Group* × *type of trial* × *language*	*5.98*	*0.006*^a^

a *The Type of Trial × Language interaction was significant only in the L1L3 LB group. The italic values are indicates that p < 0.05*.

ERPs were analyzed using four factors: Group (L1L2 EB vs. L1L3 EB vs. L1L3 LB), Type of Trial (Switch vs. No-switch), Language of Response (L1 vs. L2/L3), and Hemisphere (Left vs. Right).

Degrees of freedom were [2, 33] for the Group effect and the interactions and [1, 33] for the factors Type of trial, Language of Response, and Hemisphere.

**Table:**

	***F*-value**	***p*-value**
**GENERAL ANOVA ON THE P1 LATENCY**		
Group	0.87	0.43
Type of trial	0.75	0.39
Language of response	0.26	0.61
*Hemisphere*	*22.81*	*<0.001*^a^
Group × type of trial	0.27	0.77
Group × language of response	0.09	0.92
Group × hemisphere	0.46	0.63
Type of trial × language of response	1.81	0.19
Type of trial × hemisphere	0.001	0.98
Language of response × hemisphere	0.49	0.49
Group × type of trial × language	1.78	0.18
Group × type of trial × hemisphere	3.05	0.07
Group × language × hemisphere	1.02	0.37
Type of trial × language × hemisphere	0.39	0.54
Group × type of trial × language × hemisphere	0.83	0.44

a *The P1 was delayed over the left hemisphere. The italic values are indicates that p < 0.05*.

**Table:**

	***F*-value**	***p*-value**
**GENERAL ANOVA ON THE P1 MEAN AMPLITUDE**		
Group	1.51	0.24
Type of trial	2.25	0.14
Language of response	0.76	0.39
Hemisphere	1.56	0.22
Group × type of trial	1.27	0.29
Group × language of response	0.35	0.71
Group × hemisphere	0.56	0.58
Type of trial × language of response	0.007	0.93
Type of trial × hemisphere	0.48	0.49
Language of response × hemisphere	0.56	0.46
Group × type of trial × language	0.89	0.42
Group × type of trial × hemisphere	2.70	0.11
Group × language × hemisphere	2.66	0.09
Type of trial × language × hemisphere	0.16	0.69
Group × type of trial × language × hemisphere	0.58	0.56
**GENERAL ANOVA ON THE P2 LATENCY**		
Group	0.59	0.56
Type of trial	2.53	0.12
Language of response	3.65	0.07
Hemisphere	1.85	0.18
Group × type of trial	0.58	0.57
Group × language of response	0.08	0.92
Group × hemisphere	0.65	0.53
Type of trial × language of response	0.39	0.54
Type of trial × hemisphere	0.23	0.64
Language of response × hemisphere	0.18	0.68
Group × type of trial × language	0.04	0.97
Group × type of trial × hemisphere	2.69	0.08
Group × language × hemisphere	2.53	0.09
Type of trial × language × hemisphere	0.00	0.97
Group × type of trial × language × hemisphere	1.69	0.20
**GENERAL ANOVA ON THE P2 MEAN AMPLITUDE**		
Group	2.65	0.09
Type of trial	0.35	0.56
Language of response	0.08	0.78
*Hemisphere*	*15.39*	*<0.001*^a^
Group × type of trial	1.45	0.25
Group × language of response	2.92	0.08
Group × hemisphere	1.02	0.37
Type of trial × language of response	0.005	0.95
Type of trial × hemisphere	1.15	0.29
Language of response × hemisphere	0.08	0.78
Group × type of trial × language	2.21	0.13
Group × type of trial × hemisphere	0.87	0.43
Group × language × hemisphere	0.33	0.72
Type of trial × language × hemisphere	1.87	0.18
Group × type of trial × language × hemisphere	0.57	0.57

a *The P2 was larger over the left hemisphere. The italic values are indicates that p < 0.05*.

**Table:**

	***F*-value**	***p*-value**
**GENERAL ANOVA ON THE N2 LATENCY**		
Group	0.19	0.83
Type of trial	1.29	0.26
Language of response	1.85	0.18
Hemisphere	0.44	0.51
Group × type of trial	0.76	0.48
Group × language of response	0.84	0.44
Group × hemisphere	1.30	0.29
Type of trial × language of response	1.52	0.23
Type of trial × hemisphere	0.10	0.75
Language of response × hemisphere	0.27	0.61
Group × type of trial × language	0.31	0.74
Group × type of trial × hemisphere	0.32	0.73
Group × language × hemisphere	0.43	0.65
Type of trial × language × hemisphere	0.19	0.66
Group × type of trial × language × hemisphere	0.41	0.67
**GENERAL ANOVA ON THE N2 MEAN AMPLITUDE**		
*Group*	*3.90*	*0.03*^a^
Type of trial	0.38	0.54
Language of response	1.22	0.28
Hemisphere	0.75	0.39
Group × type of trial	0.25	0.78
Group × language of response	0.89	0.42
Group × hemisphere	0.17	0.84
Type of trial × language of response	1.22	0.28
Type of trial × hemisphere	2.90	0.10
Language of response × hemisphere	0.41	0.53
Group × type of trial × language	1.90	0.17
Group × type of trial × hemisphere	0.46	0.64
Group × language × hemisphere	0.48	0.62
Type of trial × language × hemisphere	1.10	0.30
Group × type of trial × language × hemisphere	0.07	0.93

a *The L1L3 LB group differed from both other groups in terms of N2 mean amplitude. The italic values are indicates that p < 0.05*.

**Table:**

	***F*-value**	***p*-value**
**GENERAL ANOVA ON THE LPC MEAN AMPLITUDE**		
*Group*	*3.87*	*0*.*03*^a^
*Type of trial*	*6.82*	*0*.*01*^b^
Language of response	0.59	0.45
*Hemisphere*	*12.97*	*0*.*001*^c^
Group × type of trial	0.12	0.89
Group × language of response	0.41	0.67
Group × hemisphere	1.01	0.37
*Type of trial* × *language of response*	*12.67*	*0*.*001*^b^
Type of trial × hemisphere	0.003	0.96
Language of response × hemisphere	2.30	0.14
Group × type of trial × language	0.96	0.39
Group × type of trial × hemisphere	1.90	0.17
Group × language × hemisphere	2.06	0.14
Type of trial × language × hemisphere	3.51	0.09
Group × type of trial × language × hemisphere	0.68	0.51

a *The L1L2 EB group differed from both other groups in terms of LPC mean amplitude*.

b *The LPC mean amplitude was larger in switch than in no-switch trials when responses were given in L2 and not in L1*.

c *The LPC was larger over the left hemisphere. The italic values are indicates that p < 0.05*.
